# Supplementary material for: Identification of photoperiod-induced specific miRNAs in the adrenal glands of Sunite sheep (Ovis aries)
Source: Front Vet Sci. 2022 Jul 22;9:888207. doi: 10.3389/fvets.2022.888207 (PMC9354845; doi:10.3389/fvets.2022.888207)
Supplement: Supplementary file 1 [file Data_Sheet_1.ZIP › Supplementary file/miRDeep2 file.zip!/miRDeep2 file/Know_Structure/SP42A2/oar-mir-16b.pdf]

miRBase precursor : oar-mir-16b
Total read count : 48882
oar-miR-16b read count : 48821
remaining reads : 61

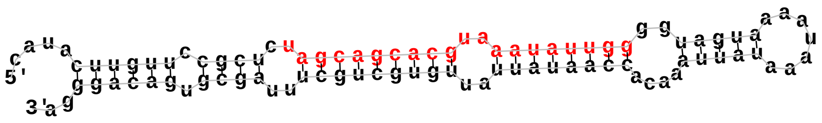

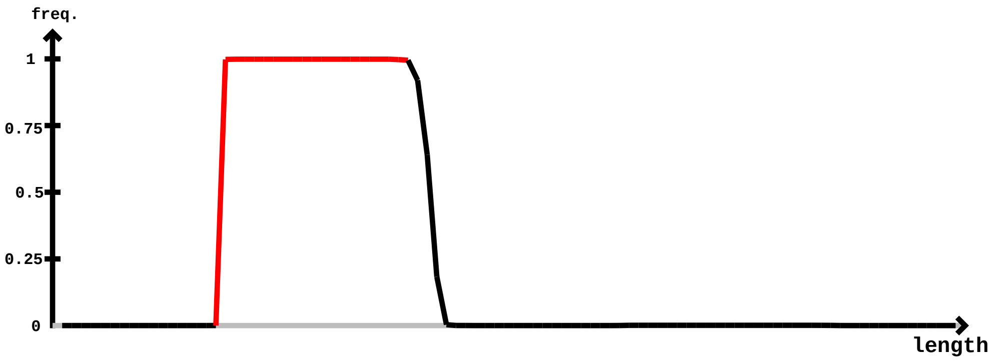


```
                             oar-miR-16b
5'- cauacuuguuccgcucuagcagcacguaaauauugggguaguaaaauaaauauuaaacaccaauauuauugugcugcuuuagcgugacaggga   -3'    exp
    ....((((((.((((..(((((((((..((((((((..(((((.......)))))....))))))))..)))))))))..)))).))))))..    reads      mm         sample
    ..............ucuagcagcacguaaauauugggg.......................................................    1          0          seq
    ...............cuagcagcacguaaauauug..........................................................    1          0          seq
    ...............cuagcagcacguaaauauugg.........................................................    3          0          seq
    ...............cuagcagcacguaaauauuggg........................................................    1          0          seq
    ...............cuagcagcacguaaauauugggg.......................................................    1          0          seq
    ...............cuagcagcacguaaauauuggggu......................................................    3          0          seq
    ...............cuagcagcacguaaauauuggggua.....................................................    1          0          seq
    ................uagcagcacguaaauauu...........................................................    82         0          seq
    ................uagcagcacguaaauauug..........................................................    109        0          seq
    ................uagcagcacguaaauauugg.........................................................    3671       0          seq
    ................uagcagcacguaaauauuggA........................................................    12786      1          seq
    ................uagcagcacguaaauauuggg........................................................    859        0          seq
    ................uagcagcacguaaauauuggAg.......................................................    20231      1          seq
    ................uagcagcacguaaauauugggg.......................................................    2101       0          seq
    ................uagcagcacguaaauauuggggu......................................................    3311       0          seq
    ................uagcagcacguaaauauuggAgu......................................................    5458       1          seq
    ................uagcagcacguaaauauuggggua.....................................................    149        0          seq
    ................uagcagcacguaaauauugggguag....................................................    7          0          seq
    ................uagcagcacguaaauauugggguagu...................................................    8          0          seq
    ................uagcagcacguaaauauugggguagua..................................................    1          0          seq
    .................agcagcacguaaauauugg.........................................................    2          0          seq
    .................agcagcacguaaauauuggA........................................................    11         1          seq
    .................agcagcacguaaauauugggg.......................................................    1          0          seq
    .................agcagcacguaaauauuggAg.......................................................    17         1          seq
    .................agcagcacguaaauauuggggu......................................................    3          0          seq
    .................agcagcacguaaauauuggAgu......................................................    3          1          seq
    ..................gcagcacguaaauauugg.........................................................    1          0          seq
    ..................gcagcacguaaauauuggAg.......................................................    5          1          seq
    ..................gcagcacguaaauauuggAgu......................................................    2          1          seq
    ..................gcagcacguaaauauuggggu......................................................    1          0          seq
    .........................................................caccaauauuauugugcugcu...............    1          0          seq
    .........................................................caccaauauuauugugcugcuu..............    3          0          seq
    .........................................................caccaauauuauugugcugcuuu.............    5          0          seq
    .........................................................caccaauauuauugugcugcuuua............    1          0          seq
    ..........................................................accaauauuauugugcugcu...............    6          0          seq
```

oar-miR-16b

```
cauacuuguuccgcucuagcagcacguaaauauuggguaguaaaauaaauauuaaacaccaauauuauugugcugcuuuagcgugacaggga
..........................................................accaauauuauugugcugcuu..............    8    0    seq
..........................................................accaauauuauugugcugcuuu.............    23   0    seq
..........................................................accaauauuauugugcugcuuua............    4    0    seq
...........................................................ccaauauuauugugcugcuuu.............    1    0    seq
```
